# Surgical Approaches for Allergic Rhinitis: A Systematic Review Protocol

**DOI:** 10.29337/ijsp.160

**Published:** 2021-08-13

**Authors:** Soumya Soumya, Gideon Adegboyega, Hassan Elhassan

**Affiliations:** 1 The University of Adelaide, AU; 2 Joanna Briggs Institute– Centre of Excellence, Adelaide, AU; 3 Flinders Medical Centre, AU; 4 Queen Mary University of London, Barts and The London School of Medicine, London, UK; 5 Homerton University Hospital, London, UK

**Keywords:** Allergic rhinitis, surgical intervention, systematic review, turbinate surgery, vidian neurectomy

## Abstract

**Background:**

Exposure to inhaled allergens in patients with allergic rhinitis results in IgE mediated hypersensitivity of nasal mucosa. The mainstay of management is allergen identification and avoidance, pharmacotherapy with antihistamines, corticosteroids and nasal douching and immunotherapy. Patients refractory to medical management can be offered surgical interventions aimed at providing symptom relief. The objective of this review is to evaluate the effectiveness of surgical intervention on functional and symptomatic outcomes in patients with allergic rhinitis that have failed medical management.

**Methods:**

Prospective and retrospective studies that assess the effectiveness of intranasal surgery to include inferior turbinate surgery, posterior nerve resection, vidian neurectomy, septoplasty and endoscopic sinus surgery (ESS) in patients that have failed medical treatment for proven allergic rhinitis. Medline, Web of Science and Embase will be searched for studies published in English from 1990. Two authors will independently screen the search results and assess the full text of potentially relevant studies. Studies that meet the inclusion criteria will be critically appraised and the data will be extracted and synthesised by two authors.

**Ethics and Dissemination:**

Ethical approval was not required for this study as secondary data will be collected. The results will be disseminated through peer-reviewed medical journal.

**Systematic Review Registration:**

This protocol has been registered on the International Prospective Register of Systematic Reviews (PROSPERO; registration number: CRD42020223773).

**Highlights:**

- Allergic rhinitis (AR) is a chronic immune-mediated inflammatory nasal condition with hallmark symptoms of sneezing, nasal obstruction, mucus discharge and anosmia in adverse cases.
- A plethora of interventions are at the hands of otolaryngologists in order to relieve symptoms of AR, however literature is yet to determine which method is best for patient outcome and quality of life.
- The primary objective of this study is to convey a systematic qualitative analysis of all the surgical interventions used in AR management.

## 1. Introduction

Allergic rhinitis (AR) is a chronic immune-mediated inflammatory nasal condition with hallmark symptoms of sneezing, nasal obstruction, mucus discharge [1] and anosmia in adverse cases. AR poses a significant burden on society and individuals [2] with an overall reported prevalence of 10%–41% depending on geographical location [3]. In AR, exposure of allergens to nasal epithelium in sensitised individuals results in the influx of inflammatory mediators. Nasal epithelial infiltration results in mucosal oedema, autonomic neural stimulation, and increased mucosal secretions that produce AR symptoms. In children, AR has been associated with rhinosinusitis, Eustachian tube dysfunction, and conductive hearing loss affecting learning and development [4]. Some studies have identified fatigue, headache, cognitive impairment and sleep disturbance as possible consequences of AR in adults [5]. The mainstay of management is allergen identification and avoidance, pharmacotherapy with antihistamines, corticosteroids and nasal douching and immunotherapy [6]. Patients refractory to medical management can be referred to otolaryngologists for consideration of surgical intervention aimed at providing symptomatic relief. Nasal obstruction is the most common refractory symptom in AR; thus the primary aim of surgical intervention is the reduction of nasal obstruction as a means of improving patient quality of life (QoL) [7].

### 1.1. Inferior Turbinate Surgery

The mucosal lining of the inferior turbinates are the first point of contact for inhaled allergens, making them a prime site for allergic inflammation and nasal obstruction. Inferior turbinate surgery increases the cross-sectional area of the nasal valve relieving the sensation of nasal blockage. Additionally, reduction in the net mucosal surface area; limits the attachment points for potential allergens [8]. This surgical technique can target the mucosal covering, submucosal layer, and a bony centre or infrequently all three components [7,9]. Submucosal resection by microdebrider or bony resection are widely used along with cautery, radiofrequency laser and coblation.

### 1.2. Vidian Neurectomy

The secretory-motor stimulus to the lacrimal gland and nasal mucosa is provided by the efferent fibres of the pterygopalatine ganglion [10]. The vidian nerve carries the preganglionic parasympathetic fibres of the greater petrosal nerve that synapses at the pterygopalatine ganglion and the postganglionic sympathetic fibres of the deep petrosal nerve. Overstimulation of this ganglion results in mucosal secretion causes rhinorrhoea. Vidian neurectomy interrupts the autonomic supply to the pterygopalatine ganglion [11].

### 1.3. Posterior Nasal Nerve Surgery

Cryosurgical ablation of the posterior nasal nerve (PNN) has the advantage of reducing the parasympathetic tone and subsequent nasal antigen response whilst minimising symptoms associated with vidian neurectomy i.e. persistent dry eye. With the addition of an endoscopic ClariFix device, the cryogen can be delivered to a specific anatomic site at a regular rate mitigating side effects [12]. Posterior nasal neurectomy involves selection resection of pathological neural networks around the inferior turbinate that cause unregulated hypersensitivity in AR. Nerve bundles containing parasympathetic and sympathetic branches of the vidian nerve are selectively cut at the level of the sphenopalatine foramen in order to provide symptomatic relief [13].

### 1.4. Septoplasty

Nasal obstruction owing to septal deviation mandates the use of septoplasty, sometimes in addition to an inferior turbinate ablation technique [14]. Although studies have conveyed meanial improvements in nasal obstruction as a result of septoplasty surgery [15], the use of this technique has demonstrated improved quality of life, particularly when used in conjunction with other interventions [16].

### 1.5. Endoscopic Sinus Surgery

Endoscopic Sinus Surgery (ESS) has become one of the most common intranasal surgical interventions. ESS is particularly efficacious in AR patients with co-morbidities such as chronic sinusitis and nasal polyposis, rather than as a primary intervention for AR. In many cases, it may be combined with inferior turbinate surgery to improve symptomatic outcome following AR surgery [16].

## 2. Objectives

### 2.1. Primary

Assess the effectiveness of surgical interventions on functional and symptomatic outcomes in patients with allergic rhinitis that have failed medical management.

### 2.2. Secondary

Outline the subsequent QoL associated with different surgical interventions.

## 3. Methods and Analysis

This protocol has been developed in accordance with the Preferred Reporting Items for Systematic Review and Meta-Analysis Protocols (PRISMA-P) guidelines. The review was prospectively registered on the PROSPERO International Prospective Register of Systematic Reviews (Registration Number: CRD42020223773). Pertinent amendments made to the protocol will be updated and published alongside the results of the systematic review.

### 3.1. Eligibility Criteria

#### Participants

The review will consider studies that include patients with failed medical management of allergic rhinitis of any age. Allergic rhinitis diagnosis should have been made with skin prick tests using standard allergen extract or by the measurement of allergen specific serum IgE by radioallergosorbent test (RAST) or enzyme linked immunoabsorbent assays (ELISA). Maximal medical management will be defined as combination of both antihistamines and nasal steroids, or immunotherapy, in addition to allergen avoidance measures in all the patients. The patient must be refractory to this management for greater than six weeks. Any studies that included participants that did not have a definitive diagnosis will be excluded unless details of individual patients that had the tests can be extracted from the author or the article.

#### Intervention(s)

This review will consider studies that will evaluate intranasal surgical interventions including inferior turbinate surgery, posterior nerve section, vidian neurectomy, septoplasty and functional endoscopic sinus surgery.

#### Comparator(s)

This review will consider studies that compare surgical intervention to medical management or studies that compare one surgical intervention versus another.

#### Outcomes

This review will consider studies that include the following outcomes: proportion of patients with symptomatic improvement in nasal patency as assessed by visual analogue score or a comparative subjective assessment scale. Similar scales assessed by the child or parent will be used for paediatric patients. Where available objective measures of nasal patency (e.g. rhinometry) or relief of other symptoms such as nasal discharge and sneezing measured by quality of life measures or other questionnaires will be considered. The follow up period will be considered short-term if <12 months and long term if >12 months.

#### Type of Study

This review will consider both experimental and quasi-experimental study designs including randomized controlled trials, non-randomized controlled trials, before and after studies and interrupted time-series studies. In addition, analytical observational studies including prospective and retrospective cohort studies, case-control studies and analytical cross-sectional studies will be considered for inclusion. This review will also consider descriptive observational study designs including case series, individual case reports and descriptive cross-sectional studies for inclusion. Studies published in insert English will be included. Studies published from database inception to the present will be included.

### 3.2. Information sources

The databases to be searched include Medline, Web of Science and Embase. Backward citation tracing will be employed in order to increase the robustness of the search strategy.

### 3.3. Search strategy

The search strategy will aim to locate both published and unpublished studies. An initial limited search was undertaken to identify articles on the topic. The text words contained in the titles and abstracts of relevant articles, and the index terms used to describe the articles were used to develop a full search strategy for Medline (see Appendix 1). The search strategy, including all identified keywords and index terms, will be adapted for each included information source. The reference list of all studies selected for critical appraisal will be screened for additional studies.

### 3.4. Data management

Following the search, all identified citations will be collated and uploaded into EndNote ×9 /2018 (Clarivate Analytics, PA, USA), duplicates removed and title & abstract as well as full text screening will take place. Full text screened articles will be extracted on a Microsoft Excel spreadsheet proforma.

### 3.5. Study selection

Titles and abstracts will then be screened by two independent reviewers for assessment against pre-defined eligibility criteria. The full text of selected citations will be assessed in detail against the inclusion criteria by two independent reviewers. Reasons for exclusion of full text studies that do not meet the inclusion criteria will be recorded and reported in the systematic review. Any disagreements that arise between the reviewers at each stage of the study selection process will be resolved through discussion, or with a third reviewer. The results of the search will be reported in full in the final systematic review and presented in a Preferred Reporting Items for Systematic Reviews and Meta-analyses (PRISMA) flow diagram [17].

### 3.6. Data Extraction

Data will be extracted from studies included in the review by two independent reviewers using a data extraction tool on MS Excel. The data extracted will include specific details about the populations, study methods, interventions, and outcomes of significance to the review objective. Any disagreements that arise between the reviewers will be resolved through discussion, or with a third reviewer. Authors of papers will be contacted to request missing or additional data, where required. In order to ensure the robustness of this extraction form, a short pilot proforma consisting of 5 studies per reviewer will take place, and additional columns will be added should pertinent themes be discovered during extraction.

### 3.7. Outcomes and Prioritisation

The outcomes of interest for this review are as follows:

1. Symptomatic and functional outcome following AR surgery
2. Patient QoL following AR surgery

### 3.8. Risk of Bias Assessment

Eligible studies will be critically appraised by two independent reviewers at the study level modify as appropriate if appraisal will occur at the outcome level for methodological quality in the review using standardized critical appraisal instruments from the Joanna Briggs Institute for experimental and quasi-experimental studies modify as appropriate if additional study designs will be included and cite the tools to be used. Authors of papers will be contacted to request missing or additional data for clarification, where required. Any disagreements that arise will be resolved through discussion, or with a third reviewer. The results of critical appraisal will be reported in narrative form and in a table.

All studies, regardless of the results of their methodological quality, will undergo data extraction and synthesis (where possible).

### 3.9. Data synthesis

A narrative approach to data synthesis will be adopted. The scope of AR surgical management will be presented. Study design, type and the comparator groups detailed in the study will be ascertained. Pertinent characteristics of the study population will be analysed particularly relating to their age, presence of risk-factors and comorbidities. Distinct surgical techniques will be assessed and subsequent patient symptomatology and functional outcome will be determined. Additionally, the QoL of patients following surgery will be noted, as well as the QoL measure used.

## 4. Ethics and Dissemination

Ethical approval was not required for this study as secondary data will be collected. The results will be disseminated through peer-reviewed medical journal.

## 5. Limitations

This study will not address studies written in languages other than English, hence an extensive amount of literature in these languages will be unaddressed in this review.

## Amendments

Amendments to this protocol will be documented

## Appendix 1

### Supplementary Figures

**Supplementary Figure 1: MEDLine Search Strategy**

1. SEASONAL ALLERGIC RHINITIS/ or ALLERGIC RHINITIS/
2. (allergic rhinitides or rhinitides or seasonal allergic rhinitides).ab,kw,ti.
3. (recurrent allergic rhinitis or refractory allergic rhinitis).ab,kw,ti.
4. SURGERY/ or SURGICAL INTERVENTION/
5. (total inferior turbinectom* or turbinectomy surgery or “inferior turbinate reduction”).ab,kw,ti.
6. (lateral outfracture or “outfractur*”).ab,kw,ti.
7. submucosal resection.ab,kw,ti.
8. (laser vaporisation or radio frequency ablation or collation or microdebrider submucosal reduction).ab,kw,ti.
9. septoplasty.ab,kw,ti.
10. (Vidian neuronectomy or posterior nasal nerve surgery or cryosurgical ablation) .ab,kw,ti.
11. (Functional endoscopic sinus surgery or “endoscopic sinus surgery”) .ab,kw,ti.
12. 1 or 2 or 3
13. 4 or 5 or 6 or 7 or 8 or 9 or 10 or 11
14. 12 and 13
15. limit 14 to yr=”1990 -Current”

## Abbreviations

AR: Allergic Rhinitis

QoL: Quality of Life

PNN: Posterior Nasal Nerve

ESS: Endoscopic Sinus Surgery

PRISMA-P: Preferred Reporting Items for Systematic Review and Meta-Analysis Protocols

RAST: Radioallergosorbent Test

ELISA: Enzyme linked immunosorbent assays
